# Autonomic and peripheral nervous system function in acute tick‐borne encephalitis

**DOI:** 10.1002/brb3.485

**Published:** 2016-05-05

**Authors:** Bernhard Neumann, Wilhelm Schulte‐Mattler, Sophie Brix, Peter Pöschl, Wolfgang Jilg, Ulrich Bogdahn, Andreas Steinbrecher, Ingo Kleiter

**Affiliations:** ^1^ Department of NeurologyUniversity Medical Centre RegensburgRegensburgGermany; ^2^ Department of NeurologyKrankenhaus der Barmherzigen Brüder RegensburgRegensburgGermany; ^3^ Department of Medical Microbiology and HygieneUniversity Medical Centre RegensburgRegensburgGermany; ^4^ Department of NeurologyHELIOS Klinikum ErfurtErfurtGermany; ^5^ Department of NeurologySt. Josef‐HospitalRuhr‐University BochumBochumGermany

**Keywords:** Autonomic nervous system, central nervous system infection, encephalitis, inflammatory neuropathy, meningitis

## Abstract

**Objectives:**

Tick‐borne encephalitis (TBE) is an emerging flaviviral zoonosis in Central and Eastern Europe. TBE can present as meningitis, meningoencephalitis, or meningoencephalomyelitis. Dysfunction of the autonomic (ANS) and peripheral motoric and sensory nervous system (PNS) might contribute to acute and long‐term complications. We aimed to examine, whether the ANS and PNS are affected in acute TBE.

**Methods:**

Fourteen patients with acute TBE, 17 with diabetic polyneuropathy (d‐PNP), and 30 healthy controls (HC) were examined in our single‐center, prospective study. ANS and PNS function was assessed by time‐ and frequency‐domain parameters of the heart rate (HR) variability at rest and deep respiration, and by sural and tibial nerve neurography. Primary endpoint was the HR variability at rest measured by root mean square of the successive differences (RMSSD). Autonomic symptoms and quality of life (QoL) were assessed by questionnaires.

**Results:**

Tick‐borne encephalitis patients had a lower RMSSD at rest (TBE 13.1 ± 7.0, HC 72.7 ± 48.3; *P* < 0.001) and deep respiration (TBE 42.8 ± 27.0, HC 109.7 ± 68.8; *P* < 0.01), an increased low‐frequency to high‐frequency power component ratio at rest (TBE 4.0 ± 4.0, HC 0.8 ± 0.5; *P* < 0.001), and a higher minimal heart rate at rest (TBE 85.4 ± 7.0, HC 69.5 ± 8.5; *P* < 0.001), all similar to patients with d‐PNP, indicating sympathovagal imbalance with increased sympathetic activation. Compared to HC, sural and tibial nerve conduction velocities and action potential amplitudes were reduced, ANS symptoms were more frequent, and QoL was lower in patients with TBE.

**Conclusions:**

The ANS and to a lesser degree the PNS are affected by acute TBE, which could potentially contribute to short‐ and long‐term morbidity.

## Introduction

Tick‐borne encephalitis (TBE) is a virus‐mediated infection, traditionally viewed to exclusively affect the central nervous system (CNS) (Dumpis et al. 1999; Kaiser 1999). It is caused by the Tick‐borne encephalitis virus (TBEV), which belongs to the family Flaviviridae, and is transmitted by infected ticks of different species. TBE is endemic in Central and Eastern Europe and parts of Asia. In the last decades its incidence has been rising in Europe, likely due to various factors including climate change, social and economic changes, and low vaccination coverage rates in endemic regions (Mansfield et al. 2009). In 2013 and 2014, 685 TBE cases were registered by the Robert Koch Institute (RKI) in Germany (Robert Koch‐Institut, 2015a).

Tick‐borne encephalitis typically takes a biphasic disease course. After a short prodromal stage, often followed by an asymptomatic interval, acute CNS symptoms occur along with high fever. The clinical presentation ranges from mild meningitis to severe meningoencephalitis or meningoencephalomyelitis (Kaiser 1999). The latter is accompanied by anterior horn or radicular involvement, causing flaccid paresis and a poliomyelitis‐like syndrome with significant morbidity and mortality (Schellinger et al. 2000; Kleiter et al. 2007; Ponfick et al. 2012). Long‐lasting and disabling sequelae occur in up to 50% of survivors (Gunther et al. 1997; Haglund et al. 1996; Kaiser 2011). The case fatality rate in Europe is approximately 1% (Haglund et al. 1996; Kaiser 2011). Diffuse brain edema and involvement of the medulla oblongata and the central portions of the brain are thought to be the main causes of mortality (Dumpis et al. 1999). Dysfunction of the autonomic nervous system (ANS), a potentially life‐threatening complication of neuroinfections, has rarely been noted to occur in TBE and might contribute to morbidity and mortality. Previously, we have observed clinical and electrodiagnostic signs of ANS dysfunction in five of eight consecutive patients with TBE, mainly presenting as upper and lower gastrointestinal tract symptoms and reduced heart rate variability (HRV) (Kleiter et al. 2006).

In this prospective study we investigated clinical and electrodiagnostic features of the ANS and the peripheral motoric and sensory nervous system (PNS) in patients with acute TBE to assess whether the ANS and PNS in addition to the CNS are affected in the course of TBE. According to our case report series (Kleiter et al. 2006), we expected that TBE patients suffer more often from autonomic disturbances than healthy controls (HC). As further control group we examined patients with long‐lasting diabetic polyneuropathy (d‐PNP).

## Methods

### Patients and study design

We conducted a single‐center, cross‐sectional, prospective study including all patients with acute TBE presenting to our tertiary referral center between August 2006 and July 2010. Inclusion criteria were male or female gender, age 18–70 years, and acute TBE defined by typical clinical symptoms, detection of anti‐TBE‐immunoglobulin (Ig)M and IgG in the serum, lymphocytic pleocytosis in the cerebrospinal fluid (CSF), and onset of neurologic symptoms within the last 14 days. Inclusion criteria for acute TBE‐matched national guidelines issued by the RKI (Robert Koch‐Institut, 2015b). TBE patients were compared to patients with d‐PNP and HC. Additional inclusion criteria for d‐PNP were type I or II diabetes with HbA1_c_ > 6.5 and polyneuropathy diagnosed according to national guidelines (Heuß et al. 2008). Exclusion criteria are shown in Table S1. Only two patients of the TBE group had premedication consisting of aspirin, clopidogrel, simvastatin, pantoprazole, bisoprolol, enalapril in one, and gabapentin in the other patient.

The primary endpoint of the study was the HRV at rest, measured by root mean square of the successive differences (RMSSD). Secondary endpoints included ANS function measured by other parameters as described in Electrodiagnostic studies and the clinical status. In addition, the presence of other autonomic symptoms and impact on quality of life (QoL) were assessed by newly designed questionnaires (Figs. S1, S2), both validated against the control groups. The QoL questionnaire was designed on the basis of the established EORTC QLQ‐C30 (Aaronson et al. 1993). Standard CSF analysis was done in our certified CSF laboratory. TBEV infection was confirmed by detection of specific IgM and IgG antibodies in serum and CSF by enzyme‐linked immunosorbent assay (Enzygnost^®^ Anti‐TBE Virus [IgG, IgM]; Siemens Healthcare Diagnostics Products GmbH [former Dade Behring], Marburg, Germany). Magnetic resonance imaging (MRI) of the brain and spinal cord was not part of the prospective study protocol, but was performed on a 1.5 Tesla system (Siemens Symphony) according to standard protocols and with contrast agent when clinically indicated.

### Electrodiagnostic studies

Electrodiagnostic procedures were done using standard equipment (Multiliner; Toennies, Höchberg, Germany) and procedures. All tests were carried out in the morning between 8 and 10 am. For at least 3 h prior to the test, no consumption of alcohol, nicotine, and caffeine was allowed. Medication with potential effects on the ANS was paused for 24 h before testing. The examination was started after the participants had rested for at least 20 min in supine position in a comfortable warm room. In TBE patients, electrodiagnostic studies were done between day 3 and 14 after onset of neurological symptoms. To exclude fever as a cause of heart rate (HR) and HRV changes, we checked the body core temperature of TBE patients with an in‐ear thermometer before testing. Ten had a temperature between 36.0 and 37.4°C, one had mild fever with a temperature of 38.1°C. In three patients the electrophysiological and autonomic testing was done after they were discharged, all of them had a temperature below 37.5°C. Before ANS testing was done, heart rhythm disorders were excluded by a 12‐lead electrocardiogram (ECG) over at least 1 min.

The investigations comprised nerve conduction studies of right sural and tibial nerves and HRV at rest for 5 min, deep respiration for at least 1 min and during the Valsalva maneuver (VM). RR intervals with an accuracy of 1 ms were recorded during normal breathing and deep breathing at 6 respirations per minute. Artifact‐free periods of at least 1 min were analyzed. To ensure a regular respiratory frequency during HR analysis at rest and deep respiration, commandos for inspiration and expiration were given. At rest the participants had to breathe in and out 12 times per minute, at deep respiration eight cycles with metronomic breathing at a frequency of 6 per minute were done. The examination of the VM was done in supine position with the upper part of the body elevated by 30°. The participants had to hold a constant pressure of 40 mmHg for at least 15 sec. The VM was repeated three times and the best attempt was taken for analysis. The RMSSD is one of many parameters for measurement of HRV and reflected ANS disturbance better than others in patients with Parkinson's disease (Maetzler et al. 2015). For spectral analyses fast Fourier transformation was applied. The very low‐frequency power component (VLF) was calculated as the power within a frequency range 0.003–0.04 Hz, the low‐frequency power component (LF) and high‐frequency power component (HF) within a range 0.04–0.15 and 0.15–0.4 Hz, respectively (Berger et al. 1986). HF is parasympathetic mediated, whereas VLF and LF correlates primarily with sympathetic activity. The calculated ratio of LF/HF and VLF/HF is considered to represent the “balance” of sympathetic and parasympathetic activity (Pagani et al. 1997). Furthermore, the difference and ratio of the HR in inspiration and expiration (E/I ratio) as well as during the VM and the maximal, minimal, and mean HR during rest and deep respiration were calculated.

### Ethics statement

The study was approved by the ethics committee of the Medical Faculty of the University of Regensburg. All participants provided written informed consent.

### Statistical analysis

Parametric electrophysiological data were analyzed by one‐way ANOVA, using the Neuwman–Keuls Multiple Comparison Test as post hoc test. Non parametric data like HRV were evaluated with the Kruskal–Wallis Test and Dunn's Multiple Comparison Test as post hoc analysis. For the autonomic questionnaire, we used the Chi‐square Test, the QoL questionnaire was evaluated with the Kruskal–Wallis Test, and the significance between the different groups later measured with the Mann–Whitney *U*‐Test. GraphPad Prism 5 (GraphPad Software, San Diego, CA) was used for statistical analysis.

## Results

### Patient characteristics and neurological findings

In total 14 patients (12 men, age 36–56 years, mean 46.1 years) with acute TBE were included (Table 1). Seven TBE patients, who presented to our hospital during the study period, did not meet inclusion criteria. In three cases the beginning of the symptoms was more than 14 days ago, one had received intravenous immunoglobulins in the last 6 weeks, one had a coinfection with *Borrelia burgdorferi*, and two had diabetes mellitus type II. TBE patients were compared to 17 patients with d‐PNP (14 men, age 33–70 years, mean 55.4 years) and 30 HC (13 men, age 23–62 years, mean 41.4 years).

**Table 1:** Demographic data

	HC (*n* = 30)	d‐PNP (*n* = 17)	TBE (*n* = 14)	*P* value
Age (years)	41.4 ± 12.4	55.4 ± 10.7*	46.1 ± 6.2	0.0004
Gender (m/f)	13/17	14/3	12/2	
Time since onset of disease	NA	17.8 ± 12.5 years	10.5 ± 3.1 days	

HC, healthy controls; d‐PNP, diabetic polyneuropathy; TBE, tick‐borne encephalitis; NA, not applicable.

Shown are mean ± SD.

ANOVA was used for statistical analysis. *P < 0.05 compared to TBE and *P* < 0.001 compared to HC in post hoc Newman–Keuls Multiple‐Comparison Test.

Clinical history and neurological assessment were obtained at admission (Table S2). Nine patients presented with meningitis, five with meningoencephalitis, and none with meningoencephalomyelitis. Thirteen patients (93%) had fever before admission and twelve (86%) were complaining about headache. Only two patients presented with nuchal rigidity or increased headache/neck pain upon inclination of the head. Reflexes were weak or absent in at least one limb in 12 (86%), and the vibration sense was reduced (≤7/8) in 4 (29%) patients, indicating impaired peripheral nerve function, although no clear clinically relevant dysfunction of the PNS was evident. An MRI of the brain was done in eight patients, and was normal in seven of them. One patient showed radiological signs of encephalitis with FLAIR‐ and T2‐hyperintense lesions in the left mesiotemporal lobe without contrast enhancement. One patient additionally had an MRI of the spinal cord which was unremarkable.

### Analysis of the heart rate variability

**Figure 1:**
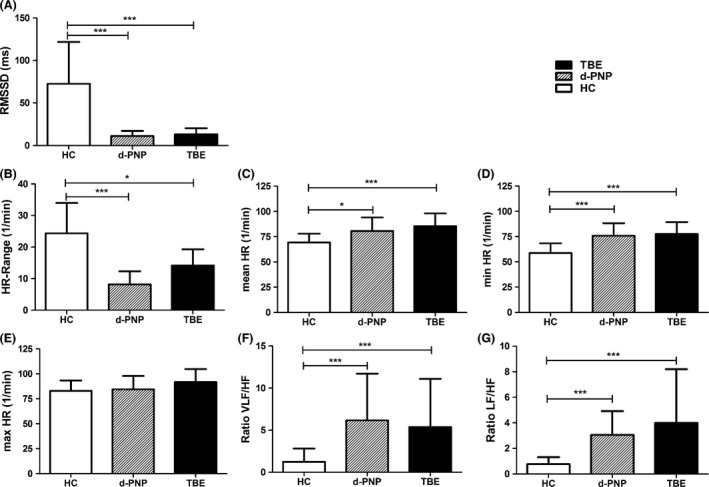
Heart rate (HR) variability at rest. HR variability at rest of healthy controls (HC;* n* = 30), patients with diabetic polyneuropathy (d‐PNP;* n* = 17), and patients with acute tick‐borne encephalitis (TBE;* n* = 14). (A) Root mean square of the successive differences (RMSSD), (B) HR range, (C) mean HR, (D) minimal HR, (E) maximal HR, (F and G) spectral analysis of HR, and calculation of very low‐frequency (VLF)/high‐frequency (HF)‐ and low‐frequency (LF)/HF ratios. Bars show mean ± SD. *P < 0.05, **P < 0.01, ***P < 0.001 (Kruskal–Wallis Test).

The primary endpoint of the study was HR variability at rest calculated by RMSSD. The RMSSD was significantly reduced in patients with TBE versus HC (Fig. 1A, Table S3) and comparable to the RMSSD in d‐PNP. Similarly, the HR range was significantly decreased and the minimal and mean HR significantly elevated in patients with TBE and d‐PNP (Fig. 1B–D). There were no changes in the maximal HR (Fig. 1E). To investigate differences in sympathetic and parasympathetic activity we performed a spectral analysis of the HRV and calculated the ratio of VLF/HF and LF/HF bands. We found a significantly increased VLF/HF and LF/HF ratio in patients with TBE and d‐PNP compared to HC (Fig. 1F and G), which, together with the lower RMSSD, indicates an increased sympathovagal balance.

**Figure 2:**
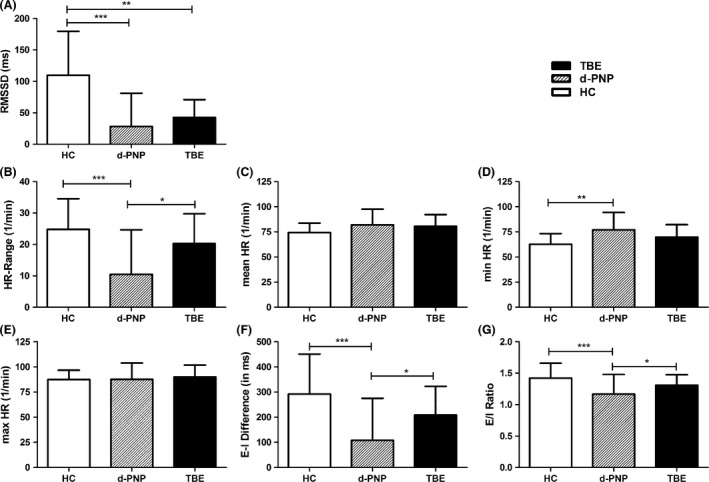
Heart rate (HR) variability at deep respiration. HR variability at deep respiration of healthy controls (HC;* n* = 30), patients with diabetic polyneuropathy (d‐PNP;* n* = 17), and patients with acute tick‐borne encephalitis (TBE;* n* = 14). (A) Root mean square of the successive differences (RMSSD), (B) HR range, (C) mean HR, (D) minimal HR, (E) maximal HR, (F) difference in the maximal RR interval during expiration and inspiration (E‐I difference), and (G) ratio of maximal RR interval during expiration and minimal RR interval during inspiration (E/I ratio). Bars show mean ± SD. *P < 0.05, **P < 0.01, ***P < 0.001 (Kruskal–Wallis Test).

Next, we evaluated the HRV at deep respiration. Again, the RMSSD was significantly lower in patients with TBE and d‐PNP compared to HC (Fig. 2A, Table S3). Although similar to HRV at rest the HR range was reduced and mean, minimal, and maximal HR were slightly elevated in TBE versus HC, none of these results reached statistical significance (Fig. 2B–E). Only patients with d‐PNP had a significant difference between the maximal RR intervals during expiration and inspiration (E‐I difference) and in the E/I ratio (Fig. 2F and G) compared to HC. Similar results were found for HRV analysis during the VM. Again, only in d‐PNP patients the HR range and the Valsalva ratio were significantly reduced (Table S3).

### Electrodiagnostic signs of polyneuropathy in patients with TBE

To confirm polyneuropathy in the d‐PNP group we performed standard neurography. We found a highly significant reduction in the nerve conduction velocity (NCV) and the motor nerve distal compound muscle action potential (CMAP) of the tibial nerve, and of the sensory nerve action potential (SNAP) of the sural nerve in patients with d‐PNP versus HC (Table 2). SNAP of the sural nerve was absent in eight patients and CMAP of the tibial nerve in five patients with d‐PNP. Surprisingly, the NCV of the tibial and sural nerve of TBE patients was also significantly reduced compared to HC (*P* = 0.039 and *P* = 0.021, respectively). Moreover, tibial nerve CMAP and sural nerve SNAP were significantly reduced, whereas the distal motor latency of the tibial nerve was unchanged. In one TBE patient a second measurement 1 week after the first test was done, which showed a further decrease in the tibial and sural NCV (Fig. S3). The electrodiagnostic tests indicated mild axonal dysfunction in TBE, criteria for demyelinating polyneuropathy were not fulfilled.

**Table 2:** Neurography of sural and tibial nerve

	HC (*n* = 30)	d‐PNP (*n* = 17)	TBE (*n* = 14)	*P* value
Sural nerve				
NCV (m/s)	54.1 ± 5.8	36.6 ± 7.8*** ^,^ ***	49.1 ± 4.6*	<0.0001
SNAP absent	0	8	0	
SNAP (*μ*V)	27.6 ± 11.6	4.8 ± 5.2*** ^,^ **	18.9 ± 8.3*	0.0429
Tibial nerve				
NCV (m/s)	49.3 ± 4.5	33.1 ± 5.0*** ^,^ ***	45.9 ± 4.4*	<0.0001
CMAP absent	0	5	0	
Distal CMAP (*μ*V)	21.6 ± 5.0	6.1 ± 5.6*** ^,^ ***	17.4 ± 5.3*	<0.0001
DML (ms)	3.9 ± 1.2	5.3 ± 1.8** ^,^ *	4.0 ± 0.6	0.0056

CMAP, compound muscle action potential; DML, distal motor latency; NCV, nerve conduction velocity, SNAP, sensory nerve action potential; TBE, Tick‐borne encephalitis; HC, healthy controls; d‐PNP, diabetic polyneuropathy.

Results are presented as mean ± SD. ANOVA was used for statistic analysis. Stars show significance in post hoc analysis with Newman–Keuls Comparison test of d‐PNP against HC (left) and TBE (right) and TBE against HC. Patients with absent SNAP (*n* = 6; all d‐PNP) and absent CMAP (*n* = 4; all d‐PNP) were excluded.

*P < 0.05, **P < 0.01, ***P < 0.001.

### Autonomic symptoms and quality of life in patients with TBE

**Figure 3:**
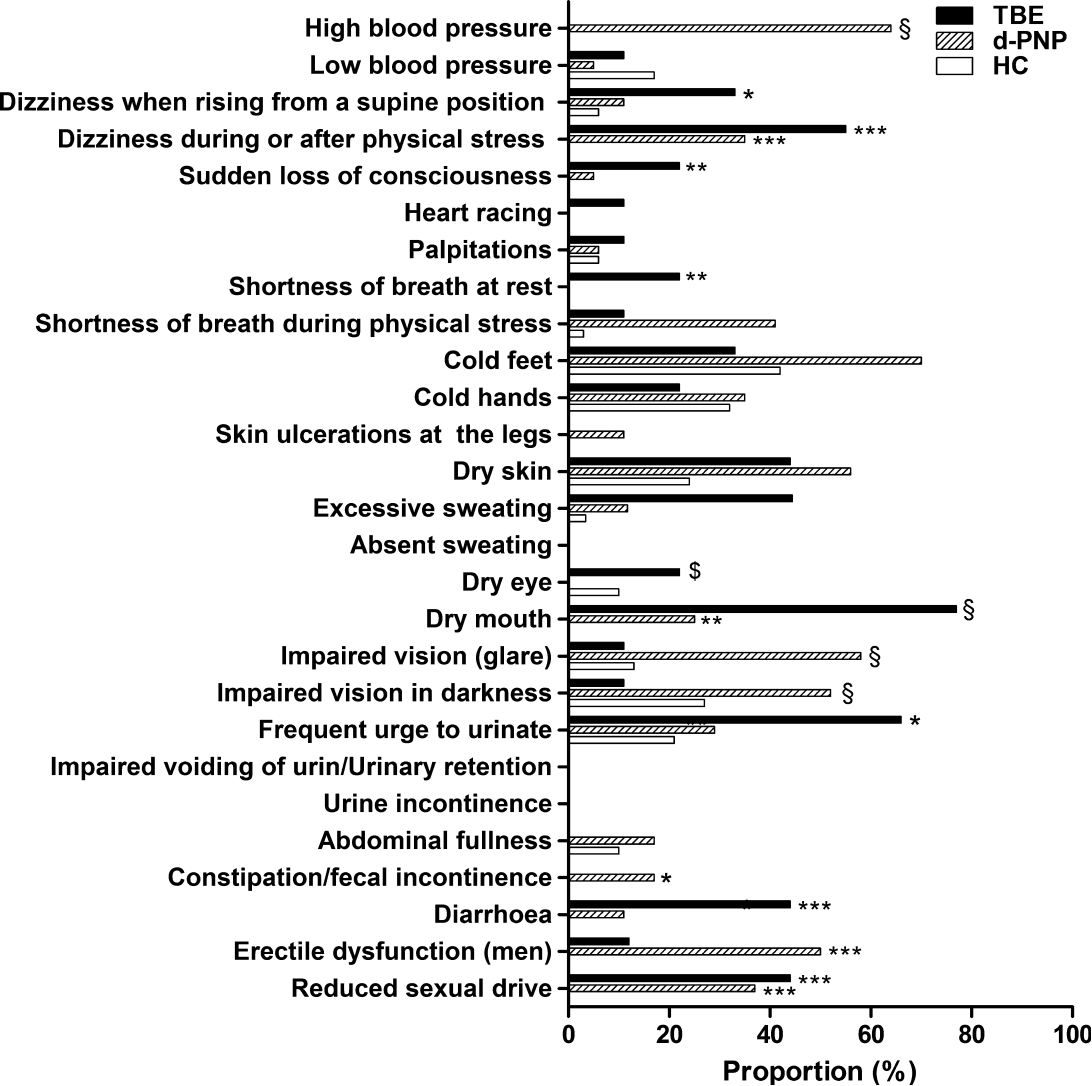
Autonomic symptoms questionnaire. Shown is the percentage of patients suffering from different autonomic symptoms, assessed with a 27‐item questionnaire, in healthy controls (HC;* n* = 29), patients with diabetic polyneuropathy (d‐PNP;* n* = 17), and patients with acute tick‐borne encephalitis (TBE;* n* = 9). *P < 0.05, **P < 0.01, ***P < 0.001 compared to HC, ^$^ *P* < 0.05 compared to d‐PNP, ^§^ *P* < 0.05 compared to both other groups (Chi‐square test).

Screening for the presence of other autonomic symptoms not captured by the electrodiagnostic tests was done by a questionnaire with 27 dichotomic items (Fig. S1). TBE patients (*n* = 9) had significantly more often orthostatic and physical exercise–induced vertigo, dry mouth, excessive sweating, frequent urge to urinate, and diarrhea compared to HC (*n* = 29) (Fig. 3, Table S4). Patients with d‐PNP (*n* = 17) had significantly more often high blood pressure, impaired vision – especially at night –, constipation, and erectile dysfunction compared to HC.

**Figure 4:**
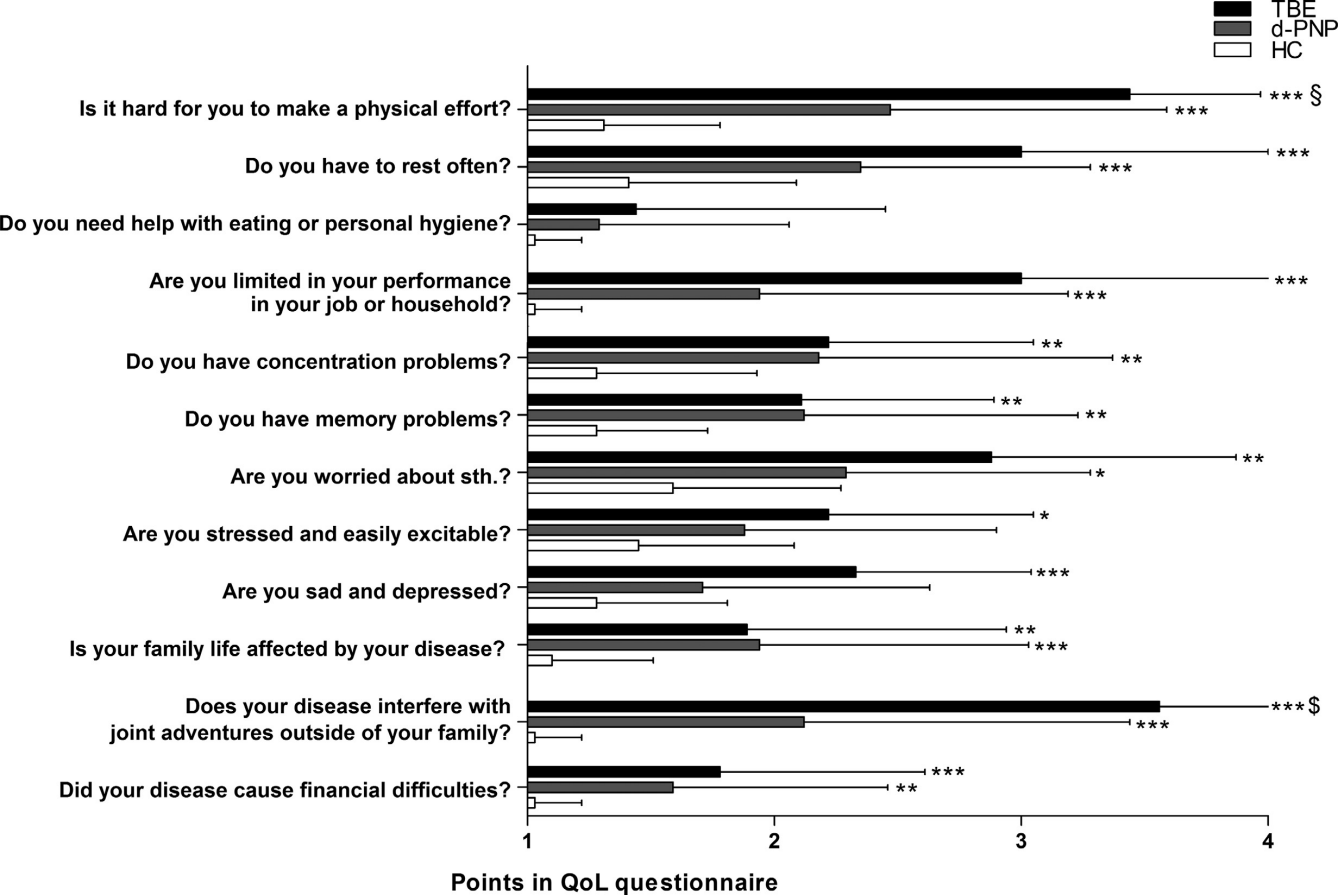
Quality‐of‐life questionnaire. Shown are the mean points on a four‐point Likert scale ± SD in healthy controls (HC;* n* = 29), patients with diabetic polyneuropathy (d‐PNP;* n* = 17), and patients with acute tick‐borne encephalitis (TBE;* n* = 9). *P < 0.05, **P < 0.01, ***P < 0.001 compared to HC, *P < 0.05 compared to d‐PNP, *P < 0.01 compared to d‐PNP (Mann–Whitney *U*‐test).

To investigate the impact of acute TBE on QoL, we asked our TBE patients to complete a standardized questionnaire (Fig. S2). In total, TBE patients (*n* = 9) had a significantly reduced QoL compared to d‐PNP and HC (Fig. 4, Table S5). TBE patients were especially limited in physical performance, but also concentration and memory were significantly impaired compared to HC.

## Discussion

In this study, we investigated clinical and electrodiagnostic features of the PNS and ANS in acute TBE. We found a reduction in both time‐ and frequency‐domain parameters of the HRV at rest and time‐domain parameters at deep respiration in patients with acute TBE. The magnitude of these alterations was similar to patients with long‐lasting d‐PNP. Furthermore, symptoms of ANS dysfunction were significantly more frequent, and QoL was significantly reduced in patients with TBE. Surprisingly, NCV and action potential amplitudes were also reduced in patients with TBE compared to HC, suggesting affliction of the PNS in acute TBE as well.

Although meningoencephalitis and myelitis, sometimes accompanied by radiculitis, are classical clinical manifestations of TBE, dysfunction of the PNS or ANS has only rarely been reported in TBE or other flaviviral infections. Previously, we have observed clinical and electrodiagnostic signs of ANS dysfunction in five patients with TBE (Kleiter et al. 2006). Other case studies described clinical symptoms of ANS dysfunction during acute disease and as persisting sequelae of TBE (Tomazic et al. 1996; Kaiser 1999; Jereb et al. 2002). It has long been recognized that about 10% of patients with TBE suffer from spinal nerve paralysis, which usually persists after recovery (Gunther et al. 1997; Schellinger et al. 2000). Interestingly, spinal nerve paralysis is not restricted to patients with myelitis, but occurs in all three clinical forms of TBE and is not correlated with the severity or duration of encephalitis (Gunther et al. 1997). Hence, it has been speculated that paralysis of spinal nerves might be a separate entity distinguished from the more common and obvious CNS manifestations (Gunther et al. 1997). Pathological evidence supporting this notion is lacking.

In this study we present for the first time an indirect electrodiagnostic proof that indeed the PNS might be involved in TBE pathophysiology. A clear correlation between reduced NCV and clinical symptoms was not seen and cannot be made because of the small sample size and the fact that some clinical symptoms could be caused by a polyneuropathy as well as the radiculitis or CNS infection, for example, absent reflexes, gait ataxia, or vertigo. Whereas infection of anterior horn neurons is a well‐recognized feature of TBE (Gelpi et al. 2005) and explains the reduction in motor NCV and CMAP amplitude, the reduction in SNAP amplitudes and sensory NCV unequivocally indicates involvement distally to the sensory ganglia, that is, unrelated to the spinal cord. Similarly, radiculitis and involvement of sensory nerves are occasionally encountered in West Nile virus infection (Jeha et al. 2003; Park et al. 2003).

Support for these clinical observations of ANS and PNS involvement in flaviviral infections comes from experimental studies. Wang et al. described autonomic symptoms, for example, distension of stomach and intestines and a reduction in the HRV, in an experimental model of hamsters infected with West Nile virus (Wang et al. 2011). Histopathological analysis in this model revealed that neuronal structures relevant for the ANS, that is, neurons in the brain stem, myenteric neurons, and cells in the sinoatrial and atrioventricular nodes were infected with West Nile virus. In a BALB/c mouse model intravenously inoculated with TBEV, viral antigens were isolated from intestinal tissues including the gastric myenteric plexus and the celiac plexus, suggesting that the virus infects the CNS via gastrointestinal autonomic nerves leading to autonomic symptoms like distension of small intestine (Nagata et al. 2015). Apart from peripheral autonomic nerves, virus antigens in this study were also found in CNS structures relevant for the ANS, for example, lumbar spinal cord, brainstem, thalamus, and hypothalamus.

We found a significantly elevated minimal HR and an increase in the VLF/HF ratio, indicating an imbalance of the sympathetic/parasympathetic cardiac innervation during acute TBE. Interestingly, the immunoactive substance sphingosine‐1‐phosphate (S1P) is increased in plasma and CSF of patients with acute TBE (Kulakowska et al. 2014). As the immune‐modulator fingolimod, which inhibits S1P signaling, leads to an increase in parasympathetic regulation in humans (Simula et al. 2015), elevation of SP1 in TBE could be an explanation for the predominant sympathetic innervations in our TBE patients.

Pathological studies from human post mortem CNS tissue revealed that the TBEV selectively affects neurons in the brainstem, cerebellum, basal ganglia, and spinal cord (Gelpi et al. 2005). Indeed, TBEV antigens were found in >50% of fatal cases in the medullar tegmentum and the pons including its raphe nuclei and the locus coeruleus, which are all parts of the central ANS. MRI shows lesions in the basal ganglia, diencephalon, cerebellum, and brainstem in approximately 20% of TBE cases (Kaiser 1999; Marjelund et al. 2004). As in our previous study (Kleiter et al. 2006), MRI of the brain failed to show specific lesions in these regions, which does not exclude TBEV infection of the central autonomic network.

An alternative explanation to direct infection of central or peripheral parts of the ANS by TBEV could be para‐/postinfectious immune‐mediated mechanisms. We previously have found that ANS dysfunction has a delayed onset, with a maximum approximately 2 weeks after the first neurological symptoms (Kleiter et al. 2006). We did not do a longitudinal analysis of ANS function in this study, but found a decrease in nerve conduction velocities in one patient in a follow‐up 1 week (day 21) after the first examination.

Although no serious cardiovascular complications in our patients occurred, we suggest monitoring of cardiorespiratory function, at least in cases with affliction of the brain stem or spinal cord. Screening for ANS function, in particular for a cardioneuropathy might help to identify patients at risk. It is well known that a TBE infection causes considerable long‐term morbidity (Haglund et al. 1996; Kaiser 2011). Whether affection of the ANS and PNS contributes to long‐term sequelae of TBE is speculative. ANS dysfunction usually recovers after acute nervous system diseases. In Guillain–Barre syndrome several reports described improvement of autonomic function irrespective of residual neuropathy (la Dornonville et al. 2005), whereas others found a clinically asymptomatic blood pressure response to standing in 27 of 33 patients (Koeppen et al. 2006).

Our study has several limitations. Autonomic testing included time‐ and frequency‐domain parameters of the HRV at 5 min of rest and of time‐domain parameters during 1 min of metronomic breathing and the VM. These tasks mainly stimulate the cardiac parasympathetic modulation. We did not perform classical cardiovascular sympathetic stimulation, for example, tilt table testing. Age in the d‐PNP group was higher and there was a male preponderance in the TBE group. It is well known that TBE shows a male predominance (Kaiser 1999), but underlying mechanisms are not yet identified. We matched the female/male ratio in TBE versus d‐PNP. Finally, the study had a small sample size and was monocentric, limiting generalization to other host or virus populations.

In summary, we show that the ANS is affected during acute TBE, potentially contributing to the diminished QoL, although it remains uncertain whether TBE in general or autonomic failure decreases QoL. Furthermore, we present evidence that the PNS is involved in acute TBE, which questions that the TBEV is only neurotropic to the CNS. Routine screening for dysfunction of the ANS and PNS as well as appropriate cardiovascular monitoring and therapy might be of relevance in the care of patients with acute TBE.

## Patient Consent

Written informed consent was obtained from all participants.

## Conflict of Interest

On behalf of all authors, the corresponding author states that there is no conflict of interest.

## Supporting information


**Table S1.** Exclusion criteria.
**Table S2.** Clinical findings of patients with TBE.
**Table S3.** Heart rate variability analysis.
**Table S4.** Results of autonomic symptoms questionnaire.
**Table S5.** Results of quality‐of‐life questionnaire.
**Figure S1.** German original and translation of autonomic symptoms questionnaire.
**Figure S2.** German original and translation of quality‐of‐life questionnaire.
**Figure S3.** Nerve conduction velocities in a patient with acute TBE.
