# Supplementary material for: Autonomic and peripheral nervous system function in acute tick‐borne encephalitis
Source: Brain Behav. 2016 May 5;6(8):e00485. doi: 10.1002/brb3.485 (PMC4864130; doi:10.1002/brb3.485)
Supplement: Supplementary file 1 — Table S1. Exclusion criteria. Table S2. Clinical findings of patients with TBE. Table S3. Heart rate variability analysis. Table S4. Results of autonomic symptoms questionnaire. Table S5. Results of quality‐of‐life questionnaire. Figure S1. German original and translation of autonomic symptoms questionnaire. Figure S2. German original and translation of quality‐of‐life questionnaire. Figure S3. Nerve conduction velocities in a patient with acute TBE. [file BRB3-6-e00485-s001.docx]

**Online supplementary material to: Neumann B, Schulte-Mattler W, Brix S, Pöschl P, Jilg W, Bogdahn U, Steinbrecher A, Kleiter I. Autonomic and peripheral nervous system function in acute tick-borne encephalitis.**

**SUPPLEMENTARY TABLES**

**Supplementary Table 1** Exclusion criteria

| For all groups | Additional exclusion criteria for HC und TBE | Additional exclusion criteria for HC |
| --- | --- | --- |
| Age below 18 or above 70  Acute infection with *borrelia burgdorferi* | Pre-existing diseases of the ANS or PNS | Regular use of other drugs than non-steroidal anti-inflammatory drugs or oral contraception |
|  | Diabetes mellitus type I or II |  |
| Infection or earlier vaccination with other flaviviruses |  |  |
| Guillan-Barré syndrome in medical history |  |  |
| Treatment with intravenous immunoglobulins in the last 6 weeks |  |  |
| Cardiac arrhythmias |  |  |
| Severe cardiac insufficiency |  |  |
| Chronic obstructive pulmonary disease |  |  |
| Acute or earlier cardiac infarction |  |  |
| Psychiatric disease (in particular alcohol abuse) |  |  |
| Solid or hematologic tumour in medical history |  |  |
| Other life threatening diseases which could impair compliance |  |  |
| Neurotoxic medications |  |  |

**Supplementary Table 2** Clinical findings of patients with TBE

| **Variable** | **Present** |
| --- | --- |
| Headache (n=14) | 12 (86%) |
| Fever (n=14) | 13 (93%) |
| Nausea (n=14) | 3 (21%) |
| Vertigo (n=14) | 8 (57%) |
| Nuchal rigidity (n=14) | 2 (14%) |
| Dysarthria or aphasia (n=14) | 4 (29%) |
| Cranial nerves impaired (n=14) | 1 (7%) |
| Sensory deficits (n=14) | 2 (14%) |
| Vibration sense impaired (≤ 7/8) (n=14) | 4 (29%) |
| Limb paresis (MRC ≤ 4) (n=14) | 3 (21%) |
| Reflexes weak or absent (n=14) | 12 (86%) |
| Coordination impaired (n=13) | 7 (54%) |
| Tandem walking impaired (n=12) | 7 (58%) |

**Supplementary Table 3** Heart rate variability analysis

|  | **HC**  (n=30) | **d-PNP**  (n=17) | **TBE**  (n=14) | **P value** |
| --- | --- | --- | --- | --- |
| HRV at rest:  RMSSD  HR range (1/min)  Mean HR (1/min)  Min HR (1/min)  Max HR (1/min)  Ratio VLF/HF  Ratio LF/HF  HRV at deep respiration:  RMSSD  HR-Range (1/min)  Mean HR (1/min)  Min HR (1/min)  Max HR (1/min)  E-I Difference (ms)  E/I-Ratio  Valsalva maneuver:  HR range (1/min)^§^  Valsalva ratio^§^ | 72.7 ± 48.3  24.3 ± 9.5  69.5 ± 8.5  58.7 ± 9.6  83.1 ± 10.1  1.2 ± 1.6  0.8 ± 0.5  109.7 ± 68.8  24.8 ± 9.6  74.4 ± 9.1  62.7 ± 10.6  87.5 ± 9.2  292.2 ± 156.4  1.4 ± 0.2  47.3 ± 19.5  1.9 ± 0.4 | 11.0 ± 5.9***  8.2 ± 4.1***  80.6 ± 13.1*  75.9 ± 12.1***  84.5 ± 13.1  6.2 ± 5.4***  3.1 ± 1.8***  28.2 ± 51.3***  10.5 ± 13.8***^/^*^$^  82.0 ± 15.1  77.1 ± 16.6**  87.7 ± 15.8  108.4 ± 162.2***/*^$^  1.2 ± 0.3***/*^$^  22.7 ± 15.5***^/^**  1.3 ± 0.2***^/^* | 13.1 ± 7.0***  14.2 ± 4.9*  85.4 ± 12.2***  77.6 ± 11.4***  91.7 ± 12.5  5.4 ± 5.5***  4.0 ± 4.0***  42.8 ± 27.0**  20.2 ± 9.1  80.6 ± 11.1  69.8 ± 11.9  90.0 ± 11.3  208.5 ± 110.2  1.3 ± 0.2  47.2 ± 14.3  1.8 ± 0.4 | <0.0001  <0.0001  0.0002  <0.0001  0.0451  <0.0001  <0.0001  <0.0001  <0.0001  0.0782  0.0048  0.3847  <0.0001  <0.0001  0.0002  <0.0001 |

Shown are the results of HRV analysis as mean ± SD. Kruskal-Wallis test was used for statistical analysis. HRV at rest and at deep respiration: Stars show significance in post-hoc analysis with Dunn`s Multiple Comparison test against HC. ^$^Except for the HR range, E-I Difference and E/I-Ratio no significant changes were found between the d-PNP and TBE groups. Valsalva maneuver: Stars show comparison of d-PNP against HC (left) and TBE (right) in Dunn`s Multiple Comparison test. ^§^TBE (n=10) and d-PNP (n=16), because 4 patients with TBE and 1 with d-PNP were not able to hold a constant pressure of 40 mmHg for at least 15 seconds. **P<0.05, **P<0.01, ***P<0.001.*

**Supplementary Table 4** Autonomic symptoms questionnaire

| **Autonomic symptom** | **HC (n=29)** | **d-PNP (n=17)** | **TBE (n=9)** | **P values chi-square-test** | | |
| --- | --- | --- | --- | --- | --- | --- |
|  |  |  |  | **HC vs. TBE** | **HC vs. d-PNP** | **TBE vs. d-PNP** |
| High blood pressure | 0 | 64.7 | 0 | NA | **<0.0001** | **0.0015** |
| Low blood pressure | 17.2 | 5.9 | 11.1 | 0.6595 | 0.2695 | 0.6341 |
| Dizziness when rising from a supine position | 6.9 | 11.8 | 33.3 | **0.0404** | 0.6564 | 0.1411 |
| Dizziness during or after physical stress (upon physical exercise) | 0 | 35.3 | 55.6 | **<0.0001** | **0.0006** | 0.3198 |
| Sudden loss of consciousness | 0 | 5.9 | 22.2 | **0.0091** | 0.1867 | 0.2147 |
| Heart racing | 0 | 0 | 11.1 | 0.0737 | NA | 0.1736 |
| Palpitations | 6.9 | 6.3 | 11.1 | 0.6821 | 0.9331 | 0.6672 |
| Shortness of breath at rest | 0 | 0 | 22.2 | **0.0091** | NA | 0.0565 |
| Shortness of breath during physical stress | 3.5 | 41.2 | 11.1 | 0.3685 | **0.0011** | 0.1141 |
| Cold feet | 42.9 | 70.6 | 33.3 | 0.6127 | 0.0706 | 0.0674 |
| Cold hands | 32.1 | 35.3 | 22.2 | 0.5711 | 0.8279 | 0.4920 |
| Skin ulcerations at the legs | 0 | 11.8 | 0 | NA | 0.0589 | 0.2842 |
| Dry skin | 24.1 | 56.3 | 44.4 | 0.1185 | 0.0312 | 0.5706 |
| Excessive sweating | 3.5 | 11.8 | 44.4 | **0.0015** | 0.2702 | 0.0599 |
| Absent sweating | 0 | 0 | 0 | NA | NA | NA |
| Dry eyes | 10.3 | 0 | 22.2 | 0.3571 | 0.1830 | **0.0493** |
| Dry mouth | 0 | 25.0 | 77.8 | **<0.0001** | **0.0055** | **0.0107** |
| Impaired vision (glare) | 13.8 | 58.8 | 11.1 | 0.8353 | **0.0014** | **0.0191** |
| Impaired vision in darkness | 27.6 | 52.9 | 11.1 | 0.3098 | **0.0214** | **0.0370** |
| Frequent urge to urinate | 21.4 | 29.4 | 66.7 | **0.0117** | 0.5457 | 0.0674 |
| Impaired voiding of urine/retention | 0 | 0 | 0 | NA | NA | NA |
| Urine incontinence | 0 | 0 | 0 | NA | NA | NA |
| Abdominal fullness | 10.3 | 17.7 | 0 | 0.3147 | 0.4478 | 0.1803 |
| Constipation/ fecal incontinence | 0 | 17.7 | 0 | NA | **0.0193** | 0.1803 |
| Diarrhoea | 0 | 11.8 | 44.4 | **0.0001** | 0.0589 | 0.0599 |
| Erectile dysfunction (men) | 0 | 50.0 | 12.5 | 0.1074 | **0.0004** | 0.0786 |
| Reduced sexual drive | 0 | 37.5 | 44.4 | **0.0002** | **0.0005** | 0.7337 |

Columns 2-4 show percentage of test persons who answered with “yes”, column 5-7 P-values calculated with the chi-square-test. P-values <0.05 are depicted in bold. NA = not applicable.

**Supplementary Table 5** Quality of life questionnaire

| **Quality of life questionnaire** | **HC (n=29)** | **d-PNP (n=17)** | **TBE (n=9)** | **P value (Kruskal-Wallis-test); all groups** | **P value**  **(Mann-Whitney-U-test);  HC vs. TBE** | **P value**  **(Mann-Whitney-U-test); HC vs.**  **d-PNP** | **P value (Mann-Whitney-U-test); TBE vs. d-PNP** |
| --- | --- | --- | --- | --- | --- | --- | --- |
| Is it hard for you to make physical effort? | 1.31  (±0.47) | 2.47  (±1.12) | 3.44  (±0.53) | **< 0.0001** | **< 0.0001** | **0.0002** | **0.0327** |
| Do you have to rest often? | 1.41  (±0.68) | 2.35  (± 0.93) | 3.00  (±1.00) | **< 0.0001** | **0.0002** | **0.0006** | 0.1032 |
| Do you need help with eating or personal hygiene? | 1.03  (±0.19) | 1.29  (±0.77) | 1.44  (±1.01) | 0.1570 | 0.0720 | 0.1030 | 0.7836 |
| Are you limited in your performance in your job or household? | 1.03  (±0.19) | 1.94  (±1.25) | 3.00  (±1.32) | **< 0.0001** | **< 0.0001** | **0.0003** | 0.0744 |
| Do you have concentration problems? | 1.28  (±0.65) | 2.18  (± 1.19) | 2.22  (±0.83) | **0.0014** | **0.0010** | **0.0039** | 0.8438 |
| Do you have memory problems? | 1.28  (±0.45) | 2.12  (± 1.11) | 2.11  (±0.78) | **0.0018** | **0.0022** | **0.0040** | 0.8209 |
| Are you worried about something? | 1.59  (±0.68) | 2.29  (± 0.99) | 2.88  (±0.99) | **0.0017** | **0.0018** | **0.0135** | 0.1720 |
| Are you stressed and easily excitable? | 1.45  (±0.63) | 1.88  (± 1.02) | 2.22  (±0.83) | **0.0358** | **0.0117** | 0.1699 | 0.2695 |
| Are you sad and depressed? | 1.28  (±0.53) | 1.71  (± 0.92) | 2.33  (±0.71) | **0.0011** | **0.0002** | 0.0822 | 0.0557 |
| Is your family life affected by your disease? | 1.10  (±0.41) | 1.94  (± 1.09) | 1.89  (±1.05) | **0.0008** | **0.0014** | **0.0005** | 0.9771 |
| Does your disease affect interfere with  joint adventures outside of your family (with friends, acquaintances)? | 1.03  (±0.19) | 2.12  (± 1.32) | 3.56  (±0.73) | **< 0.0001** | **< 0.0001** | **0.0003** | **0.0096** |
| Did your disease cause financial difficulties? | 1.03  (±0.19) | 1.59  (± 0.87) | 1.78  (±0.83) | **0.0013** | **0.0002** | **0.0036** | 0.5047 |

Columns 2-4 show mean score ± SD (score 1: not affected; score 2: little affected; score 3: moderately affected; score 4: highly affected). Columns 5-8 show statistical tests and P-values. P-values <0.05 are depicted in bold.

**SUPPLEMENTARY FIGURES**

**Supplementary Figure 1** German original and translation of autonomic symptoms questionnaire

**Original questionnaire:**

### Fragebogen autonome Symptome

Hatten Sie im letzten Monat bzw. **nach** Beginn der Erkrankung folgende Beschwerden (bitte je 1 Feld ankreuzen):

ja nein

1. Erhöhter Blutdruck 0 0

2. Erniedrigter Blutdruck 0 0

3. Schwindel / Schwarzwerden vor Augen

beim Aufstehen 0 0

4. Schwindel / Schwarzwerden vor Augen

während oder nach körperlicher Anstrengung 0 0

5. Plötzliche Ohnmacht (Synkope) 0 0

6. Herzrasen 0 0

7. „Herzstolpern“ 0 0

8. Atemnot in Ruhe 0 0

9. Atemnot bei Belastung 0 0

10. Kalte Füße 0 0

11. Kalte Hände 0 0

12. „Offene“ Beine 0 0

13. Trockene Haut (bes. Füße) 0 0

14. Übermäßiges Schwitzen 0 0

15. Fehlendes Schwitzen 0 0

16. Trockene Augen 0 0

17. Trockener Mund 0 0

18. Sehstörung durch vermehrte

Licht- / Blendeempfindlichkeit 0 0

19. Schlechteres Scharfsehen, besonders nachts 0 0

20. Häufiger Harndrang 0 0

21. Blasenentleerungsstörung / Harnverhalt 0 0

22. Harninkontinenz 0 0

23. Völlegefühl 0 0

24. Verstopfung oder Stuhlinkontinenz 0 0

25. Durchfälle 0 0

26. Erektionsstörungen (bei Männern) 0 0

27. Verminderung des sexuellen Lustempfindens 0 0

**Translation:**

### Questionnaire for autonomic symptoms

Did you have the following symptoms in the last month or **after** beginning of your disease (please make one cross for each question):

yes no

1. High blood pressure 0 0

2. Low blood pressure 0 0

3. Dizziness when rising from a supine position 0 0

4. Dizziness during or after physical stress 0 0

5. Sudden loss of consciousness 0 0

6. Heart racing 0 0

7. Palpitations 0 0

8. Shortness of breath at rest 0 0

9. Shortness of breath during physical stress 0 0

10. Cold feet 0 0

11. Cold hands 0 0

12. Skin ulcerations at the legs 0 0

13. Dry skin 0 0

14. Excessive sweating 0 0

15. Absent sweating 0 0

16. Dry eye 0 0

17. Dry mouth 0 0

18. Impaired vision (glare) 0 0

19. Impaired vision in darkness 0 0

20. Frequent urge to urinate 0 0

21. Impaired voiding of urine/ Urinary retention 0 0

22. Urinary incontinence 0 0

23. Abdominal fullness 0 0

24. Constipation / fecal incontinence 0 0

25. Diarrhoea 0 0

26. Erectile dysfunction (men) 0 0

27. Reduced sexual drive 0 0

**Supplementary Figure 2** German original and translation of quality of life questionnaire

**Original questionnaire:**

### Lebensqualität bei FSME-Patienten

Hatten Sie **in den letzten 4 Wochen** folgende Probleme (bitte je 1 Feld ankreuzen):

überhaupt wenig mäßig sehr

nicht

1. Bereitet es Ihnen Schwierigkeiten 1 2 3 4

sich körperlich anzustrengen?

2. Müssen Sie oft ruhen? 1 2 3 4

3. Brauchen Sie Hilfe beim Essen

oder der persönliche Hygiene? 1 2 3 4

4. Sind Sie bei Ihrer Arbeit

oder im Haushalt eingeschränkt? 1 2 3 4

5. Haben Sie Konzentrationsprobleme? 1 2 3 4

6. Haben Sie Gedächtnisprobleme? 1 2 3 4

7. Machen Sie sich Sorgen? 1 2 3 4

8. Sind sie angespannt und leicht reizbar? 1 2 3 4

9. Sind sie traurig und niedergeschlagen? 1 2 3 4

10. Ist durch Ihre Erkrankung Ihr

Familienleben beeinträchtigt? 1 2 3 4

11. Sind durch Ihre Erkrankung außerfamiliäre

Unternehmungen (mit Freunden oder

Bekannten) beeinträchtigt? 1 2 3 4

12. Hat Ihre Erkrankung finanzielle

Schwierigkeiten verursacht? 1 2 3 4

**Translation:**

### Quality of Life for TBE-patients

Did you have one of the following problems in the **last 4 weeks** (pleas only choose one answer each):

not at all little moderate very much

1. Is it hard for you to make a physical effort? 1 2 3 4

2. Do you have to rest often? 1 2 3 4

3. Do you need help with eating or

personal hygiene? 1 2 3 4

4. Are you limited in your performance in your job

or household? 1 2 3 4

5. Do you have concentration problems? 1 2 3 4

6. Do you have memory problems? 1 2 3 4

7. Are you worried about something? 1 2 3 4

8. Are you stressed and easily excitable? 1 2 3 4

9. Are you sad and depressed? 1 2 3 4

10. Is your family life affected

by your disease? 1 2 3 4

11. Does your disease interfere with joint adventures

outside of your family (friends, acquaintances) 1 2 3 4

12. Did your disease cause financial

difficulties? 1 2 3 4

**Supplementary Figure 3** Nerve conduction velocities in a patient with acute TBE

Shown are the nerve conduction velocities of the right tibial and sural nerve on day 14 and day 21 after onset of first neurological symptoms.
